# The Role of Child’s Age, Sex, and Temperament in Father Involvement during the Pre-School Years

**DOI:** 10.3390/children9091327

**Published:** 2022-08-31

**Authors:** Carolina Santos, Lígia Monteiro, Nuno Torres

**Affiliations:** 1 Instituto Universitário de Lisboa (ISCTE-IUL), CIS-IUL, 1649-026 Lisboa, Portugal; 2 William James Center for Research, ISPA-Instituto Universitário, 1149-041 Lisboa, Portugal

**Keywords:** father involvement, child’s age, child’s sex, child’s temperament, preschool

## Abstract

(1) Background: The aim of the study was to explore how child’s characteristics (age, sex, and temperament) were associated with father’s involvement in child-related activities. In a sample of 410 bi-parental families with pre-school age children. (2) Methods: Dividing the sample into two age groups, *OLS* regression models were conducted for each dimension of father involvement with child characteristics as predicting variables. (3) Results: for the younger children (3 and 4 years) fathers were more involved in teaching/discipline and played with their more extroverted daughters. With older children (5 and 6 years), fathers were more involved in teaching/discipline and played when children were higher on negative-affectivity. An interaction was found with boys’ higher negative-affectivity, predicting fathers’ higher involvement in teaching and discipline. (4) Conclusions: Our results suggest that children’s characteristics have an impact on what fathers do, particularly in a dimension salient to pre-school years such as teaching/discipline. This can help build tailored empirical-sustained programs aiming to encourage and support fathers’ positive involvement.

## 1. Introduction

Over past several decades, family dynamics and gender roles have undergone significant changes, with the increased participation of women in the workforce [1]. These changes have created demands but also opportunities, with fathers no longer being viewed as the main financial providers, and with the expectation that men should be more engaged in childcare and education on a daily basis [2,3]. This is paramount since, during the last few decades several studies have provided evidence that fathers do have an important role, with combined and independent effects from mothers, across different domains of child development, i.e., social competence, cognitive development, self-regulation, social adjustment, vocabulary knowledge, and quality of play (see [1,2,4,5] for review).

Parenting in general and, more specifically, father involvement is multi-determined by individual (e.g., education, parental beliefs), family (e.g., child’s characteristics, wife’s work status), social and cultural factors (e.g., social support, family’s socio-economic status) [1,6,7,8]. Several conceptual models have been proposed to systematize how these factors shape fathers, their involvement, and how they impact child development [1,6,8,9]. One important determinant of parenting is child’s characteristics, but if for some authors [10] its effects are not seen as relevant as parent’s skills and characteristics, for others [1,6,8,9] the children’s characteristics are considered as important as the parents’. Notwithstanding, a recent systematic review of the literature [11] found that out of 86 papers that met all the inclusion criteria, in the 52 that examined the determinants of father involvement, only seven looked at the effect of the child’s characteristics (e.g., age, sex, and temperament). Thus, our study aims to contribute to fill this gap by exploring the role of the child’s characteristics in shaping father involvement, in the period of the pre-school years when father-child interactions are especially salient [7], with the father being viewed as having an important role in helping the child navigate the world beyond the family, e.g., school and peers [12].

### 1.1. Child’s Age

Cabrera and colleagues’ heuristic model [1,6] considers that the effects of child’s characteristics may change over time, as children grow older, and progressively elicit more involvement from their fathers. According to [7], the father-child relationship becomes more salient during the preschool years, due to the rapid increase in children’s cognitive and socio-emotional skills and abilities, making the child a more competent and appealing partner.

During this period, the child undergoes rapid growth in terms of motor development, language, autonomy, and reasoning skills. There is a progressive development of children’s independence and autonomy in being able to accomplish important routinely daily life tasks. Whereas children around 3 are entering preschool, developing new cognitive and emotional abilities, and discovering a wider social network and social challenges; older children are working upon these competences and moving towards more sophisticated and finer tasks, e.g., with children attaining the theory of mind and executive function skills usually around 5 [13]. The quality of play also changes across the preschool years, from a more parallel to a more cooperative and symbolic play [14] promoting more complex interactions. The development of gender segregation play companions is also evident, with older children (around 5) showing a significantly greater preference for same-sex play companions than younger preschool children [15]. At this later age, children are also usually getting ready for more formal and structured learning (end of pre-school/transition to primary school), and father’s greater involvement in this age period has been associated with greater school readiness of children [16] and later academic success [17]. Some studies have found positive associations between child’s age and father’s involvement in care [18], teaching/discipline [19], and play [20], during the pre-school years, while others report none [21,22,23].

### 1.2. Child’s Sex

Regarding child’s sex, when differences are found, fathers tend to be more involved with their sons in the contexts of care and play activities [22,24,25,26,27]. It has been suggested that these differences may be related to socialization gender bias of roles and norms (see [28] for review). Another hypothesis presented by Emmott and Mace [29], based on a human behavioral ecological model, is that in Western societies, for the same fitness cost of paternal caregiving, the benefits/gains in terms of ‘child quality’ (due to the gender inequality of these societies, i.e., superior job placement, higher income, greater reproductive success), are higher for sons than daughters. Nevertheless, Pleck and Masciadrelli [30] highlight that this effect has been decreasing over time. In this sense, more recent studies have reported no significant sex differences in father’s involvement [18,19,20,23,31].

### 1.3. Child’s Temperament

Not much is known about the impact of child’s temperament on father’s parenting, with the number of studies focusing on fathers, or even mothers and fathers together, being clearly insufficient [32]. Although the issue has not received much study, some evidence has shown that fathers, more than mothers, seem to be more influenced by children’s characteristics such as temperament [33].

According to Rothbart and Ahadi [34], temperament can be described as how, due to transactions between biological predispositions and social environment, children react to stimuli and regulate their emotions and behaviors in terms of frequency and intensity across different situations. It can be summarized in three central dimensions [35]: (1) extroversion, entailing high activity and impulsivity levels, and positive expectations; (2) effortful control, referring to the ability to suppress/enact inadequate/adequate behavioral responses, and to direct and focus attention among different stimuli; and (3) negative affectivity, referring to high levels of negative feelings such as frustration, sadness, anger and discomfort, and greater feelings of shyness and difficulty to be soothed. This individual characteristic is considered to contribute to children’s adjustment due to indirect influences on parenting practices [36].

Most studies aiming to understand the relationship between child temperament and father’s involvement have analyzed temperament in terms of easy temperament vs. difficult temperament: the first described as low scores of negative effect, high positive affect scores such as extroversion; and the second associated with high scores of negative affect, low extroversion scores, and low effortful control. A child’s difficult temperament is thought to hinder father’s involvement, as it creates additional challenges for parents [37] (e.g., regarding soothing the child; managing engagement in, and transitions between activities or contexts; discipline) and is associated with higher parenting stress and low-quality interactions [38]. If some studies have reported that fathers are less involved in care with children described as having a difficult temperament [39], and in play contexts [19], suggesting that more demanding, irritable and emotional negative children might prompt less involvement then more social and easily soothed children [40], other studies have reported that fathers tend to be more involved in care [41,42] and in teaching/discipline [43] activities with these children, possibly because they exert more positive efforts, being sensible to their difficulties, than with easy-going children.

### 1.4. Interactions between Child Characteristics

It is also important to consider the possible interactions between characteristics, since child’s sex seems to play a role in the relationship between child’s temperament and father’s involvement. According to Manlove and Vernon-Feagans [44], fathers tend to be more involved with temperamentally easy sons, while other authors report that they are less involved with temperamental difficult daughters [39,45]. It has been suggested that a difficult temperament can be a potential inhibitor of the quality of parenting [46] and could exacerbate the gender bias found for father’s involvement in some samples [25,26]. However, other authors [45] have reported that fathers tended to be more involved with their difficult sons, with Feldman [47] suggesting that same-sex dyads share emotional regulation systems that might facilitate father’s engagement, even with less positive characteristics.

### 1.5. Covariates

In addition, as parenting occurs within several different social, economic, cultural, and family organization contexts [48], several sociodemographic variables have impact on father’s involvement. Fathers’ employment, and consequent demands on their available time, reduces the time they spend with their children [49]. Fathers’ working hours have been negatively associated with fathers’ involvement in direct care [22], teaching/discipline [50] and playing activities [32]. Fathers’ educational level is also an important influence [6], and has been positively associated with involvement in direct care [51], indirect care [20,24], teaching [50], play [23,52] and leisure [18,27]. It has been proposed that a father’s higher education is linked with greater availability to resources and with more knowledge about child’s needs and developmental characteristics, which in turn fosters his involvement [53].

### 1.6. Present Study

Although under-studied, enough evidence has been gathered to attest to the impact of fathers’ positive involvement on children’s socio-emotional and cognitive development [2,4,5], for review. So, it is important to better understand variables that promote or inhibit father’s involvement in child related activities on a daily basis. As less is known about how individual characteristics of the child such as sex and temperament are associated with parenting behaviors (see [11] for review) during the preschool years [54], it is important to explore which factors play a significant role during this period, and whether they foster or lessen paternal involvement.

Since studies have produced mixed results, in this study we aimed to explore how child’s characteristics such as age, sex, and temperament influence father’s involvement in different types of child-related activities. Considering two children’s age groups, i.e., 3/4 and 5/6 years old (thus differentiating developmental characteristics), we tested for the significance of sex and temperament as predictors of fathers’ involvement, although the direction of the effect is unclear. Fathers’ education and working hours were controlled for in theses analyses. Furthermore, as studies exploring the associations between temperament and fathers’ involvement reveal different associations for boys and girls [32], for review, we expected to find significant interactions between child’s temperament and sex.

## 2. Materials and Methods

### 2.1. Participants

Four-hundred and ten nuclear (i.e., married or in a civil partnership) Portuguese families with pre-school-age children were involved in the study. Fathers’ ages ranged between 24 and 56 (M = 38.26, SD = 4.90), 50.2% had primary to high school education, 49.8% had a university degree, and 96.1% were employed and worked on average 38.99 h (SD = 9.39) per week. Mothers’ ages ranged between 24 and 48 (M = 36.41, SD = 4.26), 29.8% had primary to high school education, 70.2% had a university degree, and 91.7% were employed and worked on average 34.89 h (SD = 11.80) a week. Children were divided in two groups considering the child’s age: 3–4 years (N = 118, range = 36–47.6 months, M = 42.63, SD = 3.10, 51 girls), and 5–6 years (N = 292, range = 48.23–72.17 months, M = 58.55, SD = 6.84, 161 girls). All children attended early education settings in the district of Lisbon, from which families were recruited. This was a convenience sample.

### 2.2. Instruments

Mothers completed a sociodemographic questionnaire, aiming to collect information regarding parents (e.g., age, education level, work status/hours), the child (e.g., sex, age) and family (e.g., income).

Fathers and mothers independently (order effects were controlled) completed the Parental Involvement: Care and Socialization Activities Scale [55], to assess parents’ perceptions about their participation, in relation to one another, in child-care and socialization activities occurring in everyday family-life. The scale has 26-items organized in five dimensions: direct care (five items) pertains to responsibilities regarding child’s basic needs and that require direct interaction with the child (e.g., ‘who bathes your child’); indirect care (seven items) relates to managerial and organizational tasks that ensure the child’s needs (e.g., ’who chose your child’s school’); teaching/discipline (five items) refers to the instruction of new abilities and information, and the establishment and reinforcement of rules (e.g., ‘who establishes the rules at home’); play (five items) relates to activities of play with the child (e.g., ‘who plays table-games with the child: puzzles, card-games’); and outdoor leisure (four items) refers to fun activities with the child outside the home (e.g., ‘who takes your child to the Zoo’). Both parents answered on a 5-point Likert-like scale (1—“always the mother”; 3—“both mother and father”; 5—“always the father”). In order to maximize the fidelity of the fathers’ self-reports [26], and since intra-class correlation coefficients of agreement between couples (direct care = 0.86; indirect care = 0.86; teaching/discipline = 0.67; play = 0.80; leisure outdoors = 0.82) were high, a composite value of mothers’ and fathers’ responses was calculated and used in the subsequent analyses [38,45]. All dimensions reached acceptable Cronbach’s alpha levels: direct care (α = 0.73), indirect care (α = 0.68), teaching/discipline (α = 0.71), play (α = 0.62); and outdoor leisure (α = 0.61).

The Children’s Behavior Questionnaire–Short Form Version [35,56] is a 94-item scale that allows the evaluation of child temperament as the individual manifestation of children’s reactivity and self-regulation as a result of transactions between biological factors and environment [34]. In its Portuguese validation [57], 73 of the original items were retained, maintaining the original three-factor structure: extroversion (16-items) that refers to high activity and impulsivity levels, and low inhibition (e.g., ‘always seem to be in a hurry to get from place to place’); effortful control (25-items) referring to the ability to plan, inhibit or activate responses according to the task/goal (e.g., ‘can wait to start new activities when told to wait’); and negative affectivity (32-items) which refers to the frequent experience of negative feelings such as fear, irritation, and sadness (e.g., ‘throws tantrums when he/she doesn’t get what he/she wants’). Mothers were asked to complete the questionnaire on a 7-point Likert-like scale (1—“extremely untrue of your child”; 3—“slightly untrue of your child”; 7—“extremely true of your child). All dimensions reached acceptable Cronbach’s alpha levels: extroversion (α = 0.81), effortful control (α = 0.81), negative affectivity (α = 0.83).

### 2.3. Plan of Analysis

Analyses were conducted in two steps. First, bivariate tests (product-moment correlations) were conducted to explore the inter-relationships among all the variables under study, and also assess potential multicollinearity between variables. One-way analyses of variance (*ANOVAs*) were performed to test the potential effect of the child’s sex. A second step was to conduct five multiple Ordinary Least Squares (*OLS*) regression models for each of the two age groups, with the five father involvement subscales as dependent variables (one model for each subscale) and the following variables as predictors: child’s age, sex, and temperament (extroversion, effortful control, and negative affectivity), and number of hours that fathers work, and their educational level (number of years). Additionally, the interactions terms of the child’s sex, with the three subscales of child’s temperament, were also included in all the models. The interaction effects found were explored through analysis of the simple slopes of the regression [58]. Significant predictors of non-significant regression models were reported and discussed since, statistically, these signify that even if the group of independent variables taken together as a whole do not allow a precise prediction of the dependent variable, we can still draw important conclusions about the relationships between some of the variables in the model. Statistically significant coefficients continue to represent the mean change in the dependent variable given a one-unit shift in the independent variable [59], and it is important to interpret them to avoid non-report bias [60].

## 3. Results

Initial descriptive analyses were carried out for the dimensions of father’s involvement and child’s temperament. Results are presented in Table 1. Differences regarding the child’s sex were also tested, and no significant differences were found.

Associations between father involvement, fathers’ socio-demographic covariates, and predictor variables (children’s sex, age, and temperament) were tested using Pearson correlations. In group 1, fathers’ education was positively and significantly associated with involvement in direct care (r(116) = 0.21, *p* = 0.02), indirect care (r(116) = 0.45, *p* < 0.001), teaching/discipline (r(116) = 0.21, *p* = 0.02), and outdoor leisure (r(116) = 0.24, *p* = 0.01). Children’s extroverted temperament was positively and significantly correlated with fathers’ involvement in indirect care (r(116) = 0.18, *p* = 0.049). In Group 2, fathers’ education was positively and significantly associated with involvement in direct care (r(290) = 0.14, *p* = 0.02), indirect care (r(290) = 0.19, *p* = 0.001), and play (r(290) = 0.19, *p* = 0.001). Fathers’ education was also positively and significantly associated with children’s effortful control (r(290) = 0.25, *p* < 0.001), and negatively with children’s extroversion (r(290) = −0.12, *p* = 0.047) and negative affectivity (r(290) = −0.16, *p* = 0.01). Fathers’ working hours were negatively and significantly correlated with involvement in direct care (r(290) = −0.14, *p* = 0.02) and outdoor leisure (r(290) = −0.15, *p* = 0.01).

To analyze the effects of the child’s characteristics as predictors of father’s involvement, multiple OLS regression models were conducted for each dimension of father’s involvement considering the two age groups. The summary of the models is presented in Table 2.

### 3.1. Regression Models for Group 1: 3–4 Years Old

**Figure 1:**
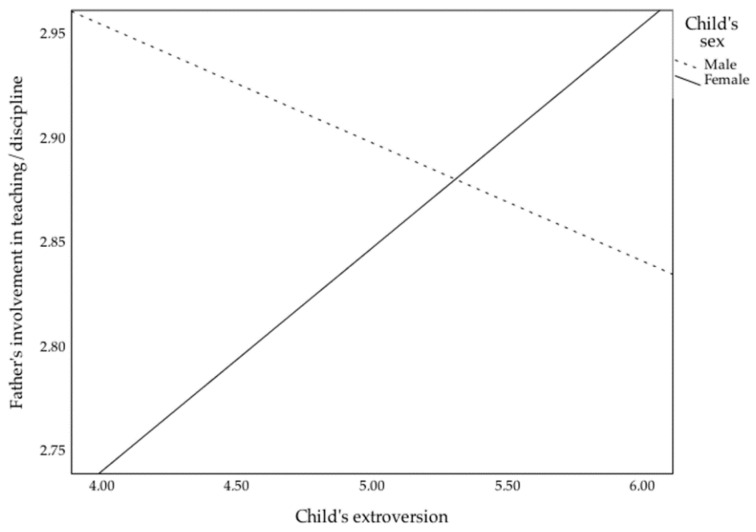
Interactions of child’s sex with child’s extroversion on father involvement in teaching/discipline, for the 3–4 years old age group.

In this age group, only the model for indirect care reached significance (F(10, 107) = 4.43, *p* < 0.001, $\eta_{p}^{2}$ = 0.29, $R_{a}^{2}$ = 0.23), with fathers’ education (*β* = 0.46, *p* < 0.001) and children’s age (*β* = −0.19, *p* = 0.04) as significant predictors. For direct care, the model did not reach significance (F(10, 107) = 1.63, *p* = 0.11), but fathers’ education (*β* = 0.22, *p* = 0.03) and the interaction between child’s effortful control and sex (*β* = 0.27, *p* = 0.04) were significant predictors. However, when analyzing the simple slopes for boys (*β* = −0.13, *p* = 0.31) and girls (*β* = 0.24, *p* = 0.10), neither was statistically significant. The model for teaching/discipline did not reach significance (F(10, 107) = 1.60, *p* = 0.12), nonetheless, the interaction between child’s extroversion and sex was found to be significant (*β* = 0.32, *p* = 0.03). The interaction term is illustrated in Figure 1. An analysis of the simple slopes showed the interaction to be significant for girls (*β* = 0.26, *p* = 0.04), but not for boys (*β* = −0.14, *p* = 0.32). The difference between the betas of boys and girls was statistically significant (*z* = −2.13, *p* = 0.02), meaning that fathers tend to be more involved in teaching/discipline with more extroverted girls, but not more extroverted boys, in this age group.

**Figure 2:**
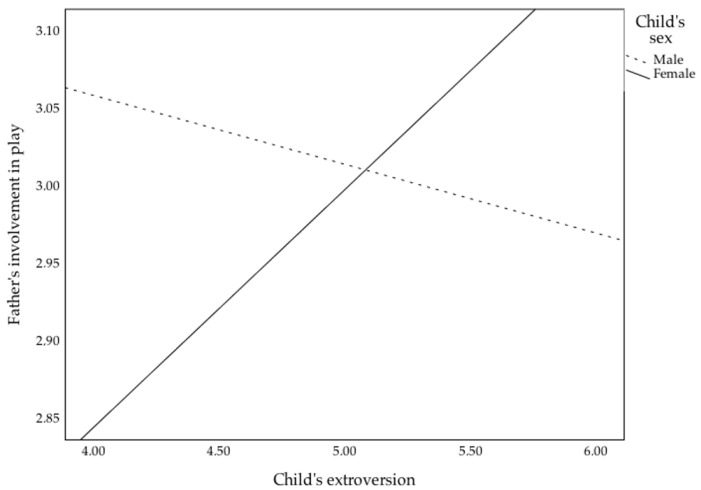
Interaction of child’s sex with child’s extroversion father involvement in play, for the 3–4 years old age group.

Similarly, the model for play was found to be non-significant (F(10, 107) = 1.25, *p* = 0.27), and only the interaction between child’s extroversion and sex attained statistical significance (*β* = 0.35, *p* = 0.02). The interaction term is illustrated in Figure 2. A simple slopes analysis revealed the interaction to be non-significant for boys (*β* = −0.10, *p* = 0.47) but significant for girls (*β* = 0.34, *p* = 0.01). The difference between the betas was statistically significant (*z* = −2.38, *p* = 0.01), meaning that fathers tend to be more involved with more extroverted girls, but not more extroverted boys in play. Finally, the model for leisure outdoors did not reach significance (F(10, 107) = 1.04, *p* = 0.41), but fathers’ education (*β* = 0.24, *p* = 0.02) was found to be a significant predictor.

### 3.2. Regression Models for Group 2: 5–6 Years Old

**Figure 3:**
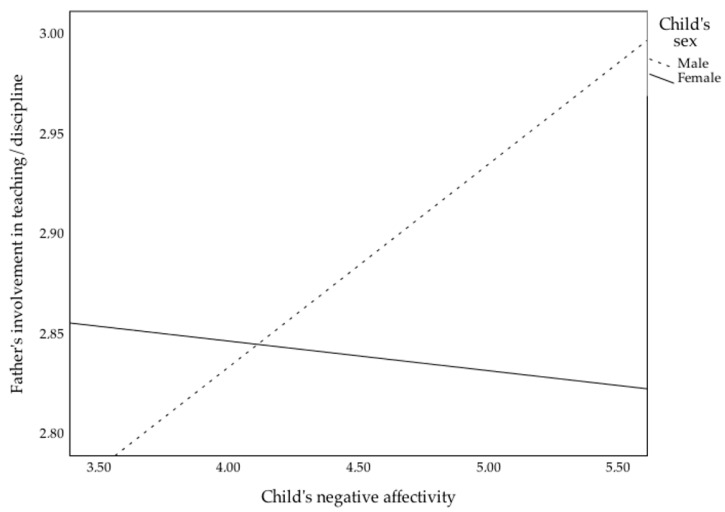
Interaction of child’s sex with child’s negative affectivity on father involvement in teaching/discipline, for the 5–6 years old age group.

Results for this age group revealed several models to be significant. Indirect care (F(10, 281) = 1.88, *p* = 0.048, $\eta_{p}^{2}$ = 0.06, $R_{a}^{2}$ = 0.03)) was statistically significant, but only fathers’ education was a significant predictor (*β* = 0.23, *p* < 0.001). The model for play was also significant (F(10, 281) = 2.09, *p* = 0.03, $\eta_{p}^{2}$ = 0.07, $R_{a}^{2}$ = 0.04), with fathers’ education (*β* = 0.22, *p* < 0.001) and child’s negative affectivity (*β* = 0.20, *p* = 0.02) being significant predictors. The teaching/discipline model was also statistically significant (F(10, 281) = 1.87, *p* = 0.049, $\eta_{p}^{2}$ = 0.06, $R_{a}^{2}$ = 0.03), with child’s negative affectivity (*β* = 0.27, *p* = 0.003), and the interaction between child’s negative affectivity and sex (*β* = −0.20, *p* = 0.02), attaining statistical significance. This interaction term is illustrated in Figure 3. An analysis of the simple slopes revealed the interaction to be significant for boys (*β* = 0.23, *p* = 0.01) but nor for girls (*β* = −0.03, *p* = 0.68); additionally the difference between the betas was statistically significant (*z* = 2.22, *p* = 0.01). Meaning that fathers tend to be more involved in teaching/discipline activities with boys who have more negative affectivity, but not with girls who have more negative affectivity.

Although the model for direct care did not reach significance (F(10, 281) = 1.73, *p* = 0.07), fathers’ education (*β* = 0.16, *p* = 0.01) and working hours (*β* = −0.16, *p* = 0.01) were significant predictors. The model for leisure outdoors was also non-significant (F(10, 281) = 1.66, *p* = 0.09), with only fathers’ working hours (*β* = −0.15, *p* = 0.01) found to be a significant predictor.

## 4. Discussion

Inconsistent results have been reported regarding the role of child’s characteristics in shaping father involvement [19,21,23,25,26,27,32,39,41,44]. Thus, the main goal of the present study was to explore how the child’s characteristics may promote or inhibit father involvement in different types of child-related activities occurring in families’ daily lives.

Results showed that for direct care, indirect care, and outdoor leisure, only sociodemographic variables (covariates) were found to be significant predictors. For the oldest group (5–6 years), fathers’ working hours were a significant predictor of involvement, that is, fathers with more overloaded work schedules participated less in direct and indirect care activities, as well as in leisure outdoors. Similar results were found in other studies in terms of associations for direct care [22,51] and leisure outdoors [50]. It is suggested that due to the higher demands of longer work-schedules, it is harder for these fathers to engage in children’s activities that follow more rigid schedules, such as feeding and bathing times [61], or require more free time such as going to the zoo or to the park. As in other studies, fathers’ education was an important predictor of his involvement, since higher levels of education are associated with the availability of greater resources and knowledge of child’s development and needs [53]. In both age groups, it was a predictor of more involvement in direct and indirect care [23,50], and for the younger children (3–4 years), of more involvement in outdoor leisure [27]. For older children (5–6 years old), it predicted more involvement in play activities [50,62].

Our results also suggest that not only children’s characteristics have an impact on the activities in which fathers are more involved (teaching/discipline and play), but also that their effects may vary as children get older [1]. For the youngest group (3–4 years old) a significant interaction was found for child’s extroversion and sex in both teaching/discipline and play activities, with fathers being more involved with their more extroverted daughters. These results are consistent with previous research findings where fathers were more engaged with more sociable daughters [39]. An extroverted temperament associated with the experience of more positive emotions and openness to the world [35] could make daughters more appealing partners to fathers, and could be a more fitting match for father’s style of interaction marked by challenging and stimulating play, while supporting children’s exploration [7,63].

For the older children (5–6 years old), children’s higher negative affectivity, which can be viewed as an inhibitor of father involvement, was a predictor of father’s higher involvement in teaching/discipline and play. Studies have reported similar associations for care and play [19,39,41], and for teaching/discipline [43]. These children might elicit a higher involvement from their fathers as they are more challenging to soothe and interact with, or fathers could respond to mothers’ perspective in the sense that they might perceive their children as being more difficult, and as such solicit more involvement from their partners [43]. An important dimension to integrate in future studies is the quality of this involvement [50,63,64,65]. That is, if it is marked by intrusiveness or an authoritarian style and restrictive practices, or if these fathers are more authoritarian and supportive, as well as sensitive and responsive, since this is expected to produce different socio-emotional outcomes for children [3,4,53]. For instance, Brown and colleagues [64,65] found that father’s sensitivity moderated the relationship between his involvement and child’s attachment security.

Additionally, for teaching/discipline, a significant interaction was found for boys’ negative affectivity. That is, fathers were more involved when their sons had higher values in the negative affectivity dimension. These results are interesting, considering the literature proposing boys as more susceptible to environmental stressors and in need of higher investment from their parents to attain healthier outcomes [66]. Furthermore, as gender identification is easier with a same-gender child, this might facilitate fathers’ involvement with their difficult sons, since their own experiences may allow for a better understatement and attunement to the child’s needs [67]. In addition, parent-infant interactive synchrony is believed to construct and regulate children’s positive arousal and affects; thus, as fathers and sons share analogous schemes of emotion regulation [47] it might be easier for them to be more involved with their more challenging sons [32].

### Limitations, Strengths and Future Research

The present study had a cross-sectional design and was based only on self-reported measures, although multiple informants were used. In the future, longitudinal studies could allow for the inference of causal relationships between father’s involvement and child’s characteristics, and for the study of bidirectionally effects [32].

Despite relying on self-reports, the study validity was increased by using distinct and independent sources to describe father participation in child-related activities. Due to high agreement, a composite measure was created. Therefore, contrary to a large number of studies, fathers’ behaviors were not described uniquely by mothers, but considering both caregivers. While studying parenting (and its impact on child development), we should consider mothers and fathers (or other significant caregivers), adopting a family-systems view such as Cabrera and colleagues [1,6] and Parke’s [8,9] models propose. As previously stated, the quality of fathers’ involvement should also be included to test for a positive father involvement [3,30].

A key strength of the study was the focus on fathers, as they are under-studied across several developmental research domains [2] and tend to be overlooked on parenting programs. Plus, this study highlighted the active role of children’s characteristics in shaping, at least in part, parenting behaviors, and therefor the need to consider these characteristics when planning empirical-sustained parenting programs.

## Figures and Tables

**Table 1:** Minimum, maximum, mean, and standard deviation of the father’s involvement and the child’s temperament dimensions.

	Total Sample			Group 1: 3–4 Years			Group 2: 5–6 Years		
	Min	Max	M(SD)	Min	Max	M(SD)	Min	Max	M(SD)
Father involvement									
Direct care	1.00	3.70	2.50 (0.52)	1.30	3.70	2.52 (0.52)	1.00	3.60	2.49 (0.52)
Indirect care	1.00	4.14	2.35 (0.43)	1.36	3.29	2.36 (0.38)	1.00	4.14	2.34 (0.45)
Teaching/Discipline	1.00	3.70	2.86 (0.31)	2.00	3.68	2.87 (0.30)	1.00	3.70	2.85 (0.45)
Play	1.80	3.90	3.03 (0.35)	2.10	3.80	3.00 (0.33)	1.80	3.90	3.04 (0.36)
Outdoor leisure	1.00	4.13	2.85 (0.42)	1.88	4.13	2.92 (0.39)	1.00	3.88	2.820 (0.43)
Child’s temperament									
Extroversion	2.00	6.75	4.94 (0.76)	2.25	6.75	4.89 (0.72)	2.00	6.63	4.96 (0.77)
Effortful control	4.00	6.88	5.59 (0.55)	4.08	6.76	5.52 (0.57)	4.00	6.88	5.61 (0.53)
Negative affectivity	2.11	6.30	4.48 (0.72)	2.22	6.30	4.53 (0.72)	2.11	6.22	4.46 (0.72)

**Table 2:** Beta (*β*) estimates of the regression models for the five dimensions of father involvement in the two age groups.

	3–4 Years					5–6 Years				
	Direct Care	Indirect Care	Teaching/ Discipline	Play	Outdoor Leisure	Direct Care	Indirect Care	Teaching/ Discipline	Play	Outdoor Leisure
	*β*	*β*	*β*	*β*	*β*	*β*	*β*	*β*	*β*	*β*
Father’s education	0.22 *	0.46 **	0.18	0.07	0.24 *	0.16 *	0.23 **	0.08	0.22 **	0.12
Father’s working hours	−0.14	−0.12	−0.04	−0.03	−0.03	−0.16 **	−0.08	−0.08	−0.04	−0.15 *
Child’s sex (1 = Feminine)	−0.05	0.04	−0.10	−0.08	−0.01	0.01	−0.07	−0.09	0.02	0.04
Child’s age	−0.10	−0.19 *	−0.14	−0.06	−0.08	−0.02	0.05	−0.10	−0.01	−0.07
Extroversion	0.07	0.17	−0.14	−0.08	0.11	0.04	0.04	−0.10	0.07	0.09
Effortful control	−0.12	0.03	−0.07	0.11	0.12	0.04	−0.03	−0.01	0.12	0.03
Negative affectivity	−0.05	0.08	0.03	0.11	0.04	−0.11	−0.02	0.27 **	0.20 *	−0.05
Extroversion × Sex	0.02	0.06	0.32 *	0.35 *	−0.19	0.01	0.02	0.05	−0.03	0.03
Effortful control × Sex	0.27 *	0.06	−0.00	−0.03	−0.10	−0.09	−0.07	0.05	−0.14	−0.01
Negative affectivity × Sex	0.20	0.05	−0.12	−0.13	0.04	0.10	0.01	−0.20 *	−0.08	−0.04
$R^{2}$	0.13	0.29 **	0.13	0.11	0.09	0.06	0.06 *	0.06 *	0.07 *	0.06
$R_{adjusted}^{2}$	0.05	0.23 **	0.05	0.02	0.00	0.03	0.03 *	0.03 *	0.04 *	0.02

* *p* < 0.05, ** *p* < 0.01.

## Data Availability

The data supporting the conclusions of this article will be made available by the authors, without undue reservation, to any qualified researcher.
